# MSWA-ResNet: Multi-Scale Wavelet Attention for Patient-Level and Interpretable Breast Cancer Histopathology Classification

**DOI:** 10.3390/jimaging12040176

**Published:** 2026-04-19

**Authors:** Ghadeer Al Sukkar, Ali Rodan, Azzam Sleit

**Affiliations:** 1 Department of Computer Science, The University of Jordan, Amman 11942, Jordan; azzam.sleit@ju.edu.jo; 2 Department of Artificial Intelligence, The University of Jordan, Amman 11942, Jordan; a.rodan@ju.edu.jo

**Keywords:** breast cancer, histopathology, deep learning, wavelet transform, attention mechanisms, explainable AI

## Abstract

Breast cancer histopathological classification is critical for diagnosis and treatment planning, yet manual assessment remains time-consuming and subject to inter-observer variability. Although deep learning approaches have advanced automated analysis, image-level data splitting may introduce data leakage, and spatial-domain architectures lack explicit multi-scale frequency modeling. This study proposes MSWA-ResNet, a Multi-Scale Wavelet Attention Residual Network that embeds recursive discrete wavelet decomposition within residual blocks to enable frequency-aware and scale-aware feature learning. The model is evaluated on the BreakHis dataset using a strict patient-level protocol with 70/30 patient-wise splitting, five-fold stratified cross-validation, ensemble prediction, and hierarchical aggregation from patch to patient level. MSWA-ResNet achieves 96% patient-level accuracy at 100×, 200×, and 400× magnifications, and 92% at 40×, with F1-scores of 0.97 and 0.94, respectively. At 200× and 400×, accuracy improves from 0.92 to 0.96 and F1-score from 0.94 to 0.97 over baseline CNNs while maintaining 11.8–12.1 M parameters and 2.5–4.8 ms inference time. Grad-CAM demonstrates improved localization of diagnostically relevant regions, indicating that explicit multi-scale frequency modeling enhances accurate and interpretable patient-level classification.

## 1. Introduction

Breast cancer is still one of the leading causes of cancer-related mortality worldwide, and early diagnosis is crucial for effective treatment and survival [1]. Histopathological analysis of biopsy samples is the gold standard for diagnosis, as it offers detailed and key information about cellular and tissue architecture features [2]. Manual analysis of histopathological slides is a complex and variable process, and these limitations have motivated the development of reliable computer-based diagnostic tools, especially in cases that are not clearly distinguishable by pathologists [3]. Recent advances in digital pathology and deep learning have enabled considerable progress in neural networks (CNNs), which effectively learn hierarchical feature representations directly from images [4], and hence, models like ResNet, DenseNet, and EfficientNet have become benchmarks due to their ability to optimize and generalize well. However, histopathological images remain challenging to classify due to large intra-class variability, small inter-class differences, high resolution, and the presence of patterns of interest across various spatial scales [5]. Conventional CNNs mostly operate in the spatial domain with fixed convolutional kernels and pooling operations, resulting in a limited capability to model frequency-based texture information explicitly. In histopathological images, a malignant pattern often exhibits complex textural irregularities across multiple scales [6], from nuclear-level details to tissue-level architecture. Thus, approaches purely based on spatial representations or spatial attention cannot capture these multi-scale and frequency-dependent characteristics.

Wavelet transforms have proven to be a very effective mechanism for multi-resolution analysis [7]; they decompose images into frequency-oriented sub-bands, preserving both spatial and spectral features. Unlike conventional downsampling, wavelet decomposition maintains high-frequency components, which are crucial for texture discrimination [8]. However, prior work has tended to use wavelets as a form of preprocessing or as static feature extractors without integrating the wavelet representation into the end-to-end deep feature learning process.

Attention mechanisms have further improved the performance of CNNs by directing models towards diagnostically relevant regions or feature channels [9], thereby enhancing the accuracy and interpretability of histopathological image analysis. However, most attention-based models operate purely in the spatial or channel domains and do not explicitly exploit frequency-domain information or adaptive scale selection, which are critical in the analysis of histopathological texture.

This paper proposes MSWA-ResNet, a multi-scale wavelet attention ResNet model designed to enhance frequency-aware representation learning in residual CNNs. The proposed model integrates recursive wavelet-guided attention mechanisms into residual blocks to support dynamic weight assignment of informative frequency sub-bands at multiple scales. The model is evaluated using a strict patient-level protocol, including stratified cross-validation, ensemble prediction, hierarchical aggregation from patch to patient level, and explainability analysis by using gradient-weighted class activation mapping (Grad-CAM) on the BreakHis dataset at multiple magnifications (40×, 100×, 200×, and 400×).

The main contributions of this paper include a new multi-scale wavelet attention mechanism, named MSWA-ResNet, which is proposed to enhance frequency-aware representation learning in residual convolutional neural networks for histopathological breast cancer image classification. MSWA-ResNet integrates recursive wavelet decomposition with residual blocks to allow for dynamic attention to informative frequency sub-bands at multiple scales. In addition, a strict patient-level evaluation protocol is adopted to prevent data leakage and ensure clinically reliable performance. Moreover, a hierarchical aggregation strategy is employed to aggregate predictions from the patch level to the image level and patient level, allowing for meaningful evaluation. Finally, model interpretability is provided using Grad-CAM visualization to highlight diagnostically relevant regions of interest in histopathological images for diagnosis. The proposed model is evaluated on the BreakHis dataset and demonstrates consistent performance improvements over baseline convolutional neural network models.

The rest of this paper is organized as follows. A review of various histopathological image classification methods, including CNN-based, attention-based, multi-scale, and wavelet-driven, is introduced. Section 2 describes the proposed model, including MSWA-ResNet architecture, dataset preparation, training strategy, hierarchical aggregation, and evaluation metrics. Section 3 reports experimental results and comparisons with other baseline and state-of-the-art models. Section 4 concludes the paper and provides directions for future work.

Current research on breast cancer histopathological image classification has increasingly focused on convolutional neural networks, attention mechanisms, multi-scale learning strategies, and wavelet-based approaches to improve feature representation and diagnostic performance.

Convolutional neural networks have emerged as the dominant approach for histopathological image classification due to their inherent ability to learn hierarchical feature representations directly from raw images. In the context of breast cancer histopathology, CNN-based approaches have demonstrated strong performance in modeling complex morphological and textural patterns associated with benign and malignant tissues. Early studies in this domain established the effectiveness of learned texture features for cancer diagnosis tasks, showing that deep convolutional architectures can automatically extract discriminative representations without reliance on handcrafted features. Subsequent developments have refined these architectures through deeper residual designs, dense connectivity, transfer learning strategies, and advanced optimization techniques, further improving classification accuracy on benchmark datasets such as BreakHis.

The authors of [10] suggested a novel framework using AlexNet as a starting point to leverage transfer learning and significant data augmentation techniques, reaching patient-level accuracy of 92.52% at 200× for the BreakHis dataset. An enhanced DenseNet-121 architecture, called IDSNet, with the addition of the squeeze and excitation attention mechanism proposed in [11], achieves accuracy between 84% and 90%, demonstrating successful recognition performance. [12] presented AHoNet, which coupled channel attention modules and second-order pooling with a ResNet model, demonstrating classification accuracy of 99.29% at 200× magnification. The authors of [13] designed a model using inception-based region-level classification along with rule-level fusion, reporting reasonable results with accuracy rates of 92% across all 40×, 100×, 200×, and 400× magnifications. The authors of [14] proposed a CNN feature fusion approach for breast cancer histopathological image classification on the BreakHis dataset using VGG16 and Resnet50 as feature extractors, achieving 93.2% accuracy at 400×. The authors of [15] proposed the cellSage model, a lightweight CNN that uses multi-resolution feature extraction and CBAM attention for the classification of breast cancer in the BreakHis dataset, yielding 94.8% accuracy at the patient level.

Although recent CNN fusion and residual-based models have improved accuracy, they often result in a lack of interpretability, efficiency, and multi-scale adaptability. Most rely primarily on either spatial-domain feature extraction or shallow fusion strategies, limiting their ability to capture the inherently multi-scale frequency-dependent characteristics of histopathological images.

Attention mechanisms have been widely used to improve the performance of CNN models by directing the network to attend to diagnostically relevant regions or feature channels, thus achieving high classification accuracy and interpretability in medical images. The authors of [16] introduced the RDTNet model, which integrates the residual deformable transformer layer into a CNN network, achieving 96.41% at 40× patient-level accuracy. The authors of [17] combined multiple pre-trained CNN models and convolutional block attention mechanisms (CBAMs) to achieve high classification accuracy above 99% on the BreakHis dataset. Similarly, [18] presented the MDAA model, integrating DenseNet and dual-adaptive attention mechanisms along with multiple-scale features, demonstrating high accuracy on the BACH and BreakHis datasets. Furthermore, [19] introduced the BCMNet model, embedding CBAM in the VGG16 model, achieving 92.2% at 200×, and [20] enhanced DenseNet by integrating improved channel and spatial attention, achieving high accuracy. Other attention-based models include M2S2-FNet, which combines attention with knowledge-sharing methods designed for multi-class classification [21], and the CSAResNet & DAMCNN ensemble models of [22], which primarily focus on channel and spatial attention by using fixed-scale feature maps. Additionally, there is the attention and detail information aggregation network named ADBNet of [23]. However, the existing attention-based models have not leveraged the frequency domain or scale features, which are essential for handling the complex textural variations observed in histopathology images.

Multi-scale learning is critical in the analysis of histopathological images, as a relevant diagnostic structure exists at different spatial scales. Several studies have introduced designs for learning models that combine different scales to enhance the robustness of classification tasks. MSI-MFNet, proposed by [24], integrates spatial and non-linear information from images at different resolutions, achieving 98% accuracy in classifying the BreakHis dataset at 200× magnification. The authors of [25] introduced MultiNet by integrating features from various pre-trained CNNs. Another proposed model [26] combined handcrafted descriptors and deep features for classification, while MPIFR, proposed by [27], used global average pooling to integrate features from different scales extracted from four CNNs and achieved 97.77% accuracy for multi-class classification. Although these models have proven the effectiveness of feature fusion from different scales, most are based on static methods of fusion or manually designed network structures. The information about scales is generally pooled together instead of being adaptively and dynamically determined.

Wavelet transforms have been increasingly used in medical image analysis tasks due to their potential for preserving spatial and frequency information across different scales. In histopathological imaging, for example, wavelet transforms have been found useful for capturing texture patterns associated with malignancy. Recently, [28] proposed DWNAT-Net, which uses discrete wavelet transform and neighborhood attention transformers, and achieved 99.66% image-level and 99.69% patient-level accuracy at 40× magnification in the BreakHis dataset. The authors of [29] proposed a lightweight CNN model designed by incorporating Haar wavelets and invertible residual modules, which achieved 99.8% image-level accuracy on BreakHis. The authors of [30] designed a model integrating wavelet texture features along with a gradient boost classifier to demonstrate stable performance across different magnifications. The authors of [31] designed a model incorporating adaptive wavelet thresholding to enhance denoising and classification. Despite their capabilities, most wavelet-based methods use wavelet transforms as preprocessing tools, feature extraction modules designed using prior knowledge, and shallow fusion modules. These methods did not implement wavelet-driven modules directly within the layers of CNN models for end-to-end learning or the fusion of wavelet representations using attention mechanisms that adapt dynamically.

Despite significant progress in histopathological image classification using CNNs, attention mechanisms, multi-scale learning strategies, and wavelet-based techniques, several limitations remain. The existing CNN models primarily operate in the spatial domain and lack explicit awareness of frequencies. Attention mechanisms are beneficial for interpretability, but they are limited to spatial or channel refinement. Multi-scale learning is limited to static feature fusion, rather than dynamic scale selection. Wavelet-based methods only are used as a preprocessor or shallow feature extractor, rather than as part of a complete deep learning architecture. Furthermore, many state-of-the-art results are obtained with a relaxed evaluation protocol that does not enforce a strict splitting of patients between the training and testing sets, suggesting a potential for data leakage. These gaps indicate a need for a unified model that combines all these important aspects, including frequency awareness, multi-scale learning, interpretability, and a strict patient-level evaluation protocol.

Recent developments in the analysis of histopathological images have also focused on multi-scale CNN approaches and attention mechanisms to better represent features and improve the performance of the classifier. For example, multi-scale CNN approaches have shown the significance of representing hierarchical contextual features at different magnification scales, and attention mechanisms have also been incorporated to better select features, particularly those relevant for diagnosis. Transformer-based approaches have shown promising performance in medical image analysis tasks, especially by utilizing self-attention mechanisms to model global features in images [32]. Even better performance has been observed using hybrid approaches that combine CNN and Transformer modules for histopathology image classification tasks [33]. However, most of these approaches have mainly focused on the spatial domain, without considering the importance of the frequency domain, which is crucial for representing texture features in images.

Unlike existing wavelet-based and attention-based approaches, the proposed MSWA-ResNet introduces a unified and tightly integrated frequency-aware learning mechanism within the residual network architecture. Prior wavelet-based methods, such as DWNAT-Net [28] and related approaches, typically employ discrete wavelet transforms as a preprocessing step or as a separate feature extraction module, where the extracted frequency features are subsequently processed by convolutional or transformer-based networks. In contrast, the proposed MSWA module embeds recursive wavelet decomposition directly into intermediate feature maps within residual blocks, enabling end-to-end optimization of frequency representations alongside spatial features during network training.

Furthermore, conventional attention mechanisms, including SE and CBAM, primarily operate in the spatial and channel domains by reweighting feature maps based on global pooling or convolutional operations, without explicitly modeling frequency-domain characteristics. In contrast, the proposed multi-scale wavelet attention mechanism performs dynamic sub-band selection based on energy responses across multiple decomposition levels, allowing the network to adaptively emphasize diagnostically relevant frequency components. This results in a frequency-aware attention process that is inherently scale-sensitive and better aligned with the multi-resolution nature of histopathological structures.

In addition, unlike many existing studies that rely on image-level data splitting or handcrafted preprocessing pipelines, the proposed framework integrates frequency-aware representation learning with a strict patient-level evaluation protocol and hierarchical aggregation strategy. This combination enables not only improved classification performance but also a more clinically reliable and interpretable diagnostic framework. Through this integration of recursive wavelet decomposition, dynamic frequency attention, and patient-level modeling, MSWA-ResNet provides a distinct methodological contribution beyond existing wavelet-enhanced or attention-based CNN architectures.

To overcome all these challenges, the MSWA-ResNet model, which integrates a recursive multi-scale wavelet attention mechanism into a residual CNN architecture, is proposed.

## 2. Materials and Methods

**Figure 1:**
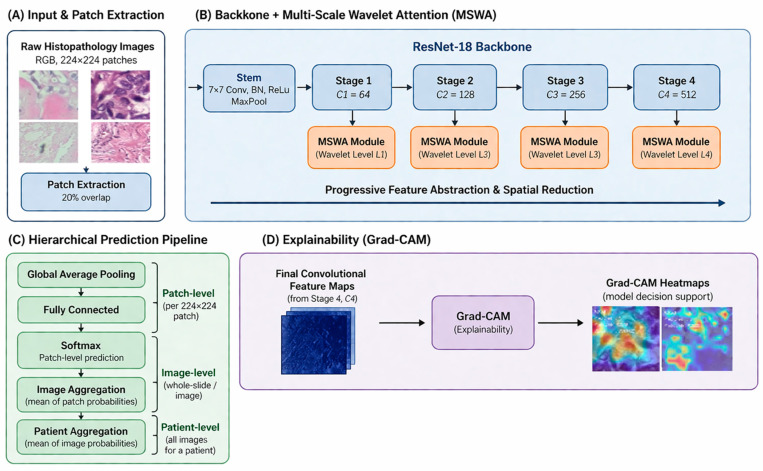
Multi-scale wavelet attention ResNet (MSWA ResNet).

**Figure 2:**
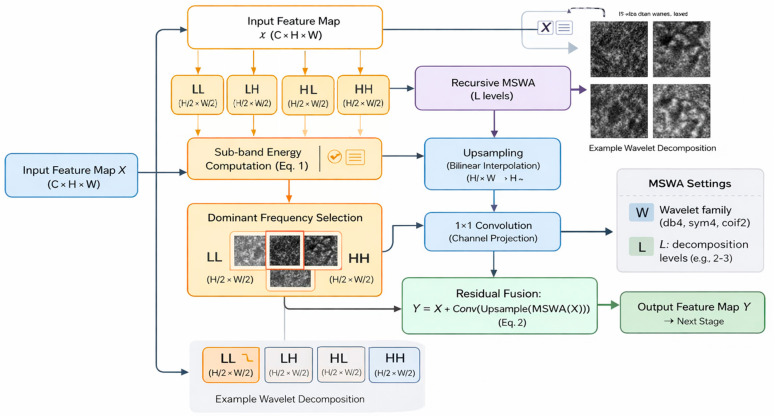
MSWA (multi-scale wavelet attention) block.

This paper proposes MSWA-ResNet, a multi-scale wavelet attention ResNet, with Explainable AI, for clinically reliable histopathology image classification at the patient level. The overall framework of the proposed model is based on the combination of patch-based learning, multi-scale wavelet attention, ensemble modeling, and visual explainability, as shown in Figure 1 and Figure 2. Whole-slide histopathology images at a given magnification are decomposed into fixed-size RGB patches of 224 × 224 pixels, with a 20% spatial overlap between adjacent patches to ensure the continuity of local morphological features. A strict patient-level splitting strategy is adopted, ensuring that there is no data leakage and the evaluation remains unbiased. The training set is divided using a stratified five-fold cross-validation, where all patches of a patient are only assigned to one fold of the cross-validation. In each experiment, four folds are used for training and validation, while the remaining fold is for testing. This approach reflets is a more practical scenario in real-world deployment, where a model must generalize to unseen patients, not unseen patches. For each fold, an independent MSWA-ResNet model is trained, leading to the training of five models with parameter values {θ_1_, θ_2_, …, θ_5_}. The predictions are made at the patient level after aggregation from the image level and patch level using ensemble averaging. Grad-CAM is used to provide explanations for the predictions made by the model. The proposed model is designed to ensure the accuracy, robustness, interpretability, and flexibility of the models for different levels of magnification, wavelet scales, and attention depth. Figure 1 illustrates the overall architecture of the proposed MSWA-ResNet framework, including patch extraction, multi-scale wavelet attention integration, and hierarchical aggregation. Figure 2 presents the internal structure of the MSWA module, highlighting wavelet decomposition, sub-band selection, and attention-based feature refinement.

### 2.1. Dataset Description

**Figure 3:**
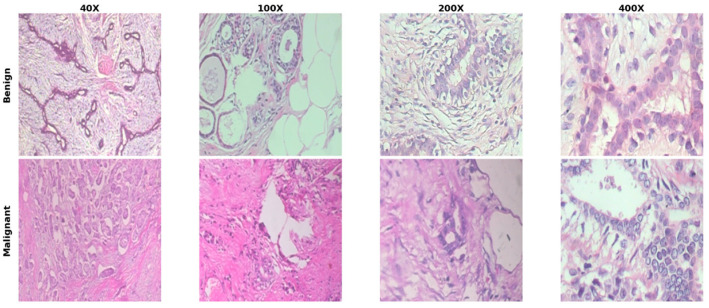
A sample of the images from the BreakHis dataset.

In this paper, the BreakHis (Breast Cancer Histopathological Image) dataset [34] has been used. It is a publicly available source of data that has been widely utilized for evaluating computer-assisted systems for breast cancer histopathological images. The dataset contains hematoxylin and eosin (H&E) stained images associated with breast biopsies of different patients, specifically 82 patients, with original image dimensions of 700 × 460 pixels (RGB). Images are provided at four different magnification scales: 40×, 100×, 200×, and 400×, allowing the models to work with images of varying spatial resolution. Images are also classified as benign or malignant, and these ground truths are provided at the patient level. More than one image exists per patient, as might be expected in real-world environments. Figure 3 shows a sample of the images from the BreakHis dataset:

In the experimentation, each test of magnification level is done independently to eliminate differences in the scale of features throughout the process. The patient information from the dataset is used throughout the experimentation process to test splitting on each patient. The BreakHis dataset has a large intra-class variation along with high patient variability, which makes it a suitable challenge for testing the robustness, generalization capability, and applicability of the MSWA-ResNet model. Table 1 shows the image-level summary of the BreakHis dataset:

### 2.2. Data Preparation and Patch Extraction

The BreakHis dataset was first divided at the patient level into 70% for training and 30% for independent testing. This ensured a strict separation of patients and avoided any data leakage. From the 70% training split, a stratified 5-fold patient-level cross-validation scheme was used for model selection. In this process, for every fold, four splits were used for training and one split was used for validation. This ensured that the benign/malignant ratio was maintained for every fold. After the model selection process, the final model was retrained on the entire training split and tested once on the independent 30% test split. All the final performance metrics reported in this paper are based on the independent test set. The composition of the dataset after splitting is summarized in Table 2.

### 2.3. Dataset Distribution and Evaluation Clarification

To further clarify the reliability of the reported evaluation metrics, it is important to emphasize that all performance measures are computed based on the aggregation of predictions across all available samples within each magnification setting, rather than being limited by the number of patients alone. Specifically, predictions are first generated at the patch level, then aggregated to the image level, and finally to the patient level using probability averaging.

As a result, although the number of patients may vary across magnification subsets, the evaluation metrics are derived from a sufficiently large number of patch- and image-level predictions, ensuring statistical stability. This aggregation strategy mitigates the impact of small patient counts in certain magnification settings and provides a more reliable estimate of model performance.

To overcome this issue, all evaluation metrics such as accuracy, precision, recall, and F1-score are calculated based on aggregated predictions for all available samples per magnification setting rather than being limited to each patient in a specific setting. This ensures that all evaluation metrics are statistically valid and are not constrained by the limited number of patients in a specific set.

Moreover, considering that the evaluation metrics are aggregated per patient and that cross-validation along with ensemble methods are applied, this minimizes the effect of class imbalance and small sample sizes in the dataset, ensuring a more robust evaluation of the model’s performance.

**Figure 4:**
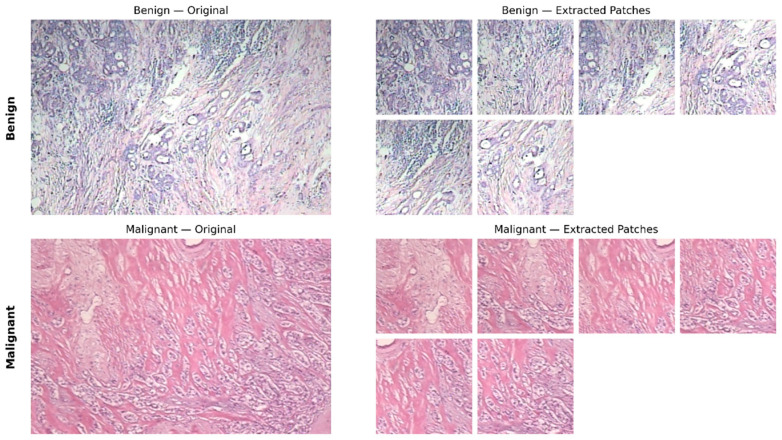
Sample of patches extracted from the original histopathological image (40×: original image vs. extracted patches (224 × 224)).

After the splitting of the dataset, each image was divided into fixed-size patches of 224 × 224 pixels with a 20% overlap. The patch extraction was carried out after the patient-level split to ensure that the patches from the same patient were not distributed across different subsets. It should be noted that the patch extraction was carried out using a valid region strategy without padding, and hence patches that did not completely fit the image boundary are excluded. Therefore, the actual number of patches for the given image may be less than the maximum obtained from the stride calculation, and hence six valid patches are extracted per image, as illustrated in Figure 4. The exact computation is detailed below.

It is important to clarify the number of extracted patches per image. Given the original image size of 700×460 pixels, a patch size of 224×224, and a 20% overlap, the stride is computed as follows:

stride=224×(1−0.20)=179 pixels.

The number of patches along each spatial dimension is determined using a valid region strategy (without padding), as follows:

$$N_{H}=\left\lfloor \frac{700-224}{179}\right\rfloor +1=\left\lfloor \frac{476}{179}\right\rfloor +1=2+1=3,$$

$$N_{W}=\left\lfloor \frac{460-224}{179}\right\rfloor +1=\left\lfloor \frac{236}{179}\right\rfloor +1=1+1=2.$$

Therefore, the total number of patches per image is

$$N=N_{H}\times N_{W}=3\times 2=6.$$

This explains why only six valid patches are extracted per image, rather than eight. The discrepancy arises because the valid region strategy excludes incomplete boundary patches that do not fully fit within the image dimensions.

Table 3 below shows the composition of the patch-level dataset, which includes the fixed test set and the average distribution of the training and validation patches from the 5-fold cross-validation process. Examples of the patches are shown in Figure 4.

To address class imbalance at the patch level, a class balancing strategy was applied within each training fold. While the benign class was augmented using geometric transformations (horizontal and vertical flipping, and rotations of 90° and 180°), the malignant class was randomly downsampled when necessary to match the size of the augmented benign class. This combined strategy ensures balanced class distributions while preventing the over-representation of the majority class.

Consequently, the final number of training patches per class reflects the balanced subset size, which may be lower than the original malignant patch count reported prior to augmentation (Table 3). This explains the observed differences between Table 3 and Table 4.

### 2.4. MSWA-ResNet Architecture

#### 2.4.1. Backbone Network

The proposed model employs ResNet-18 as the backbone network, considering its favorable trade-off between capacity and computational efficiency. ResNet-18 is based on residual learning with the addition of identity shortcut connections, which avoid the vanishing gradient problem and enable the stable training of deeper architectures. The architecture begins with the initial stem and then the four stages of residual blocks. The stem block consists of the 7 × 7 convolution with a stride of 2, followed by batch normalization, ReLU, and the 3 × 3 max pooling layer. The four stages of residual blocks have channel dimensions that increase from 64, 128, 256, and 512, respectively, represented as $C_{1}=64,\;C_{2}=128{,\;C}_{3}=256,{\;and\;C}_{4}=512$. Each residual stage consists of stacked convolutional layers with skip connections, allowing the network to learn hierarchical feature representations from low-level texture patterns to high-level semantic structures relevant for histopathological analysis. The backbone network is pre-trained with ImageNet weights for faster convergence and better generalization. A global average pooling layer is also used before the classification layers, which provides a solid foundation for integrating the proposed MSWA model.

#### 2.4.2. Multi-Scale Wavelet Attention (MSWA)

To enhance sensitivity to diagnostically relevant structures in histopathological images, the proposed model integrates a multi-scale wavelet attention (MSWA) mechanism operating in the frequency domain and integrated into a convolutional backbone. Given an intermediate feature map X produced by a residual stage of the backbone network, a discrete wavelet transform is applied using a selected wavelet family. A discrete wavelet transform is employed on feature map X, and a wavelet family is selected to decompose the feature map into four sub-bands, which are low–low (*LL*), low–high (*LH*), high–low (*HL*), and high–high (*HH*), providing information about different frequencies and orientations. The selection of wavelet families for different magnification levels was motivated by the natural multi-resolution characteristics of histopathological images, in which distinct spatial frequencies demand specific decomposition filters to optimize feature extraction efficiency. Hence, both coarse structural patterns and fine textural details can be modeled. The energy of each sub-band is computed for each level of decomposition, reflecting its relative importance. These energies are then used to guide the attention mechanism, enabling the network to selectively emphasize informative frequency components across multiple scales while suppressing less informative features. Through recursive wavelet-based attention, the MSWA module enables dynamic refinement of frequency-aware features in conjunction with spatial convolutional learning. For each level of decomposition, the sub-bands’ energies are calculated using the following equation:

(1) $$\begin{array}{cccc} & \varepsilon_{s}=\frac{1}{HW}\sum_{i=1}^{H}\sum_{j=1}^{W}X_{s}(i,j),s\in \{LL,LH,HL,HH\} & & \end{array}$$

For each level of the sub-band decomposition process, the sub-band with the highest energy is chosen as the most informative frequency component at that level of resolution, where $X_{s}(i,j)$ denotes the wavelet coefficient at the spatial position $\left(i,j\right)$ in the sub-band s, where s∈{LL,LH,HL,HH}, and H and W denote the height and width of the corresponding sub-band. The term $\varepsilon_{s}$ represents the average response of the sub-band s, obtained by averaging all the coefficients in the sub-band. The selection process is recursively applied to the sub-band with the highest energy to form the frequency-aware attention path without the need for additional spatial downsampling. The selected sub-band is upsampled to the spatial dimensions of the original feature map and is fed into a 1 × 1 convolution to ensure consistency with the number of channels. The improved frequency-aware representation is combined with the original feature map via a residual connection given by

(2) $$\begin{array}{cccc} & Y=X+{\mathrm{Conv}}_{1\times 1}(\mathrm{Upsample}(\mathrm{MSWA}(X))) & & \end{array}$$

where X is the input feature map, and Y denotes the output after wavelet-guided attention refinement. The MSWA module is inserted after each residual stage in the backbone network. This allows for the refinement of the feature representation at multiple frequency scales, increasing sensitivity to the textural irregularities and structural patterns often found in malignant histopathology images.

The MSWA module operates by explicitly linking the frequency-domain analysis in Equation (1) to the feature refinement in Equation (2). Given an input feature map $X\in R^{C\times H\times W}$, the discrete wavelet transform decomposes it into four sub-bands (LL, LH, HL, HH), each with reduced spatial dimensions of H/2×W/2. The energy of each sub-band is computed using Equation (1), and the sub-band with the highest response is selected as the dominant frequency component. This selection is recursively applied for multi-level decomposition to capture hierarchical frequency information.

The selected sub-band is then upsampled to match the original spatial resolution. In this work, bilinear interpolation is used for upsampling due to its efficiency and stability. A 1 × 1 convolution is applied to align channel dimensions before combining it with the original feature map through residual addition, as defined in Equation (2).

The MSWA module is inserted after each residual stage of the backbone network, where the feature map dimensions follow $C_{1}=64$, $C_{2}=128$, $C_{3}=256$, and $C_{4}=512$, with spatial resolution progressively reduced across stages. This enables frequency-aware refinement at multiple levels of abstraction.

#### 2.4.3. Training Strategy and Ensemble Learning

The proposed model is trained using a strict five-fold patient-level cross-validation method to ensure the accurate estimation of model performance without any leakage of patches between the patients. All the patches of the same patient are exclusively allocated to a single fold. In the cross-validation process, for every fold k ∈ {1, …, 5}, four folds are used for training and validation, and the remaining fold is allocated for testing. For every fold, a separate MSWA-ResNet model is trained using the supervised learning method along with the cross-entropy loss function. The parameters of the model are tuned using backpropagation. The model parameters are selected based on the highest performance on the validation set. The proposed model is trained on the entire dataset, and the process is repeated five times. The trained models are represented as {$\theta_{1}$, $\theta_{2}$, …, $\theta_{5}$ }. During inference, ensemble learning is adopted to enhance generalization and reduce variance. The class probability vector is calculated by the softmax function for a given patch by each model. The ensemble probability is calculated by averaging the predicted probabilities of the patches across all the folds:

(3) $${\hat{p}}_{c}(x)=\;\frac{1}{5}\sum_{k=1}^{5}p_{k,c}(x)$$

where (x) is the predicted probability for class c from the k-th model. In this way, ensemble averaging helps to minimize the variance across folds, as well as potential bias that may be introduced by fold-specific data characteristics. The approach is patch-based, followed by hierarchical aggregation to produce predictions at the patch, image, and patient levels.

To produce predictions, ensemble probabilities are first calculated for each patch. Let $p_{i,j}$ be the predicted probability of the j-th patch in i-th image and let $N_{i}$ be the number of patches in i-th image. The prediction for each i-th image is given by averaging probabilities for all patches in that image. The image-level prediction probability is given by:

(4) $${\hat{p}}_{i}\;=\frac{1}{N_{i}}\sum_{j=1}^{N_{i}}p_{i,j}$$

**Figure 5:**
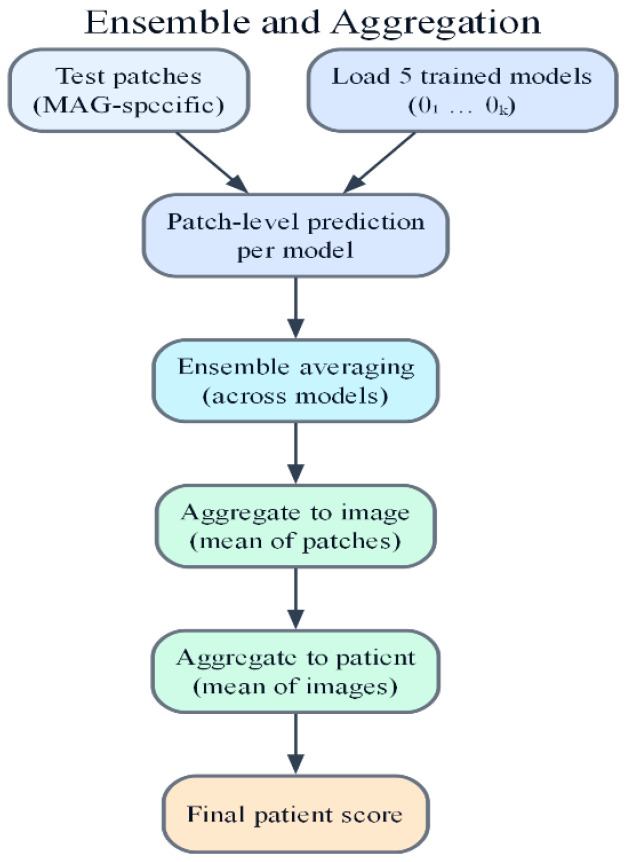
Hierarchical aggregation method.

Subsequently, patient-level predictions are computed by averaging the probabilities of all images of the same patient. The aggregation of predictions in two stages eliminates noise present in each patch, resulting in a more consistent prediction of the final diagnosis. The overall hierarchical aggregation is depicted in Figure 5. For a patient with images, the patient-level probability is:

(5) $$p_{patient}=\frac{1}{M}\sum_{i=1}^{M}p_{i}$$

Finally, the class label for the patient level is determined based on the decision threshold of 0.5, which is applied to the patient-level probability. It should be noted here that the decision strategy based on the threshold of 0.5 corresponds well with the diagnostic process, as the class label is determined based on the evaluation of all the tissue regions, as opposed to specific patches. The hierarchical aggregation framework facilitates the evaluation of the model’s performance at the patch, image, and patient levels.

#### 2.4.4. Evaluation Metrics

The performance of the proposed model is evaluated on three different levels of hierarchy: patch, image, and patient levels. The metrics used to assess the performance of MSWA-ResNet are accuracy, sensitivity, precision, and F1-score. The emphasis of the proposed model is on sensitivity, as it is a critical parameter in the classification of cancer. The accuracy of the proposed model is estimated using a 95% confidence interval, calculated using the bootstrap method of resampling with replacement. The metrics are computed based on the ensemble of the proposed model, and the decision is made based on the threshold.

Accuracy: It is the proportion of total correct predictions made by the model out of all predictions. It evaluates how often the model is correct overall, regardless of class.

(6) $$Accuracy=\frac{Correct\;classifications}{Total\;classification}\frac{TP+TN}{TP+TN+FP+FN}\;\;\;\;\;\;\;\;\;\;$$

Precision: It measures the proportion of true positive outcomes out of all positive predictions. High precision means fewer false positives, which is especially desirable in clinical diagnosis to avoid unwanted interventions.

(7) $$Precision=\frac{Correctly\;classified\;actual\;positives}{Everything\;classified\;as\;positive}\frac{TP}{TP+FP}$$

Recall: It measures how accurately the model identifies true positive cases; it is also known as the true positive rate (TPR). It is crucial to avoid false negatives in diagnostic detection.

(8) $$Recall\;\left(or\;TPR\right)=\frac{Correctly\;classified\;actual\;prositives}{All\;actual\;positives}\frac{TP}{TP+FN}$$

Specificity: It measures the model’s ability to correctly identify negative cases, alongside recall, and gives a balanced view of diagnostic performance.

(9) $$Specifity=\frac{Number\;of\;true\;negatives}{Number\;of\;true\;negatives+Number\;of\;false\;positives\;}$$

F1-Score: The harmonic mean of precision and recall, giving one measure that balances false positives and false negatives. It is especially useful when dealing with class imbalance.

(10) $$F1\;Score=2\times \frac{Precision\times Recall}{Precision+Recall}$$

**Figure 6:**
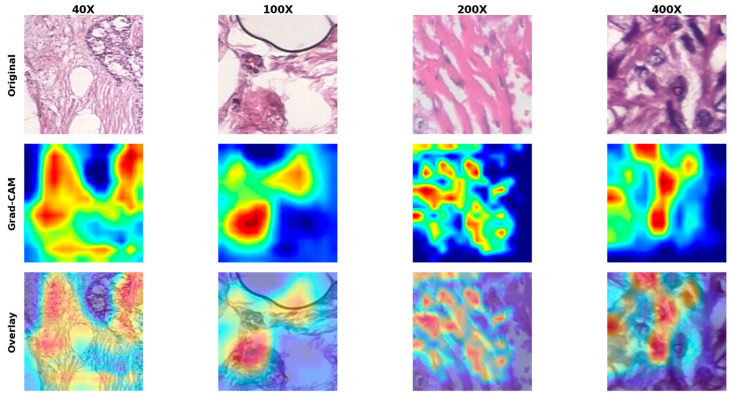
Sample of malignant patches: the original, Grad-CAM, and Overlay MSWA-ResNet.

Explainability: To improve the transparency of the model and facilitate clinical interpretability, the proposed model employs a post hoc method, gradient-weighted class activation mapping (Grad-CAM). Specifically, Grad-CAM is used for the final convolutional layer of the residual stage in the ResNet backbone. For each input patch and class, the gradients of the class score with respect to the selected feature maps are computed and globally averaged to produce the channel-wise importance weights. Then, the class activation map is computed as follows:

(11) $$\mathrm{CAM}\;=\;\mathrm{ReLU}\;(\sum_{C}\;\alpha_{C}\;A_{c})$$

where $A_{c}$ denotes the activation map of the channel and $\alpha_{c}$ is the weight derived from the gradient. The ReLU operation filters out the positive contribution. The activation map is normalized and resized to the original patch size, then overlaid on the input image to highlight the regions that contributed most to the decision made by the network. Figure 6 shows the Grad-CAM visualizations, which highlight regions of interest. From a diagnostic perspective, it can be noted that the highlighted regions are associated with histopathological structures commonly associated with malignant patterns. This includes irregular cell morphology, dense nuclei, and abnormal tissue structures. This shows that the model’s attention is focused on clinically relevant features rather than background artifacts, which supports the interpretability and reliability of the proposed approach.

### 2.5. Hierarchical Prediction and Ensemble Strategy

To guarantee clinically significant evaluation and avoid bias from patch-level predictions, a hierarchical prediction framework is proposed, which consists of three prediction levels: patch-level inference, image-level aggregation, and patient-level decision-making. At the patch level, a histopathological image is segmented into overlapping patches of a fixed size, and then the proposed MSWA-ResNet model processes each patch individually to obtain class probability scores.

At the image level, a probability averaging strategy is applied to aggregate all prediction scores from the patches of an image, and then the maximum score in the prediction vector determines the image-level label. At the patient level, a probability averaging strategy is applied to aggregate all image-level prediction scores of a patient, and then the maximum score in the prediction vector determines the patient-level label.

In addition, to improve the robustness and generalization of the proposed MSWA-ResNet model, an ensemble learning strategy is proposed, where an average prediction is computed by aggregating prediction scores from various models trained in different cross-validation folds, and then the final prediction is computed by averaging prediction probabilities from all models in the ensemble system.

### 2.6. Implementation Details

All experiments were conducted on a workstation equipped with an NVIDIA GPU (NVIDIA GeForce RTX 5070 Ti Laptop GPU (12 GB VRAM)), an Intel(R) Core(TM) Ultra 9 275HX, and 63 GB of RAM, running on a Windows 11 (10.0.26200) operating system, with CUDA version 12.8.

The training process for the model was done using the AdamW optimizer with an initial learning rate of 3 × 10^−4^. A batch size of 32 was utilized for the entire experiment, and the model was trained for 50 epochs. The training was done using the cross-entropy loss function.

The model’s parameters were initialized using the pre-trained weights from the ImageNet dataset for the ResNet-18 architecture and were expanded to include multi-scale wavelet attention (MSWA) blocks at various stages of the network. An L-level wavelet decomposition was implemented for the MSWA block, and the wavelet basis was chosen depending on the magnification level.

Five-fold cross-validation was implemented for the evaluation of the model at the patient level. The final predictions for the model were made by ensembling the five trained models by averaging the probabilities for the output. The predictions were made hierarchically, from the patch level to the image level and then to the patient level, using the mean probability aggregation method. The performance evaluation for the model was done by using the accuracy, precision, recall, and F1-score metrics, and the confidence interval for these metrics was computed using the bootstrap resampling method.

## 3. Results

To evaluate the proposed MSWA-ResNet model, performance was evaluated at the patch, image, and patient levels using a hierarchical aggregation strategy (Patch → Image → Patient). The model’s performance was found to be 0.96 for 100×, 200×, and 400× magnification, and 0.92 for 40× magnification. The F1-score was found to be 0.9714 for 100×, 200×, and 400×, and 0.9412 for 40× magnification. The 95% confidence intervals for patient-level accuracy were found to be between 0.84 and 0.88 (lower bound) and 1.00 (upper bound).

### 3.1. Best MSWA Configuration per Magnification

Table 5 describes the best-performing MSWA-ResNet configuration for each magnification level based on patient-level performance. The optimal wavelet configuration was determined empirically for each magnification level using validation results from the cross-validation folds. Specifically, the best-performing configurations are sym4 with two decomposition levels for 40×, coif3 with two levels for 100×, bior3.5 with two levels for 200×, and db4 with two levels for 400×.

These findings indicate that the optimal wavelet family and decomposition depth are magnification-dependent, reflecting the varying scale of morphological structures in histopathological images. This scale-dependent behavior emphasizes the effectiveness of incorporating multi-scale wavelet attention in the backbone network and demonstrates the flexibility of the MSWA-ResNet model in capturing magnification-specific histopathological features.

### 3.2. Comparison with Baseline and Recent State-of-the-Art Methods

In order to assess the efficacy of the proposed MSWA-ResNet in a comprehensive manner, its performance is evaluated in relation to both traditional convolutional neural network (CNN) baselines and other representative architectures that are typically utilized in recent studies on histopathological image classification tasks. These baselines include various forms of multi-scale CNN architectures and attention-based CNN architectures that are focused on incorporating hierarchical features and context-aware features in the classification process. While a direct comparison with recent studies that utilized Transformer-based or hybrid architectures is limited by variations in evaluation protocols and the lack of standardized patient-level splits in recent studies, the baselines utilized in this study provide a fair evaluation in a similar experimental setting.

MSWA-ResNet is evaluated in comparison to conventional CNN architectures like ResNet18, ResNet34, ResNet50, DenseNet121, and EfficientNet-B0, using the same patient-level data split and cross-validation settings to ensure a fair comparative analysis.

All the baseline CNN architectures are fine-tuned using the same experimental setup. The experimental results demonstrate that the proposed MSWA-ResNet model performs competitively or even surpasses the conventional CNN architectures across all magnifications, while maintaining consistent and stable performance across evaluation folds. In particular, the proposed model achieves stable patient-level accuracy of 0.96 at 100×, 200×, and 400× magnification settings, coupled with F1-scores of 0.97, while achieving competitive performance at 40× magnification settings, where the accuracy of MSWA-ResNet is found to be 0.92. From the experimental analysis, it can be concluded that the proposed model achieves superior performance by leveraging the frequency-aware multi-scale wavelet attention module in the context of discriminative feature learning, without increasing the computational complexity of the model, comparable to standard CNN architectures. Table 6 shows the performance of the cross-validation of the MSWA-ResNet model, while Table 7 and Table 8 present the comparison of patient-level performance of the proposed method with the baseline CNN backbones and the proposed MSWA-ResNet on the BreakHis dataset.

Considering the clinical importance of identifying malignant lesions, sensitivity (SEN) is regarded as one of the major evaluation criteria for the model, as reflected in the current manuscript. Sensitivity has been reported for all tables to give a more accurate clinical assessment.

The performance gains achieved by the proposed MSWA-ResNet model compared to conventional architectures can be explained by its ability to incorporate explicit frequency domain information along with spatial domain features, which may not be captured by conventional CNN architectures or even attention-based models. Unlike typical convolutional approaches, which are mainly based on the use of spatial feature hierarchies, and unlike attention mechanisms, which are generally based on channel-wise and spatial feature recalibration, the proposed model makes use of recursive multi-scale frequency decomposition within the network, allowing for the joint learning of spatial and frequency representations.

The performance gain achieved by the MSWA-ResNet model, as compared to the baseline architectures, is due to the fact that the model is able to effectively learn and incorporate frequency information, as opposed to typical convolutional and attention mechanisms, where frequency information is not addressed at all. Unlike recent developments, which are mainly based on increasing the depth of the network and employing global attention mechanisms, the proposed method is based on enhancing the features using recursive multi-scale frequency decomposition, allowing for the effective discrimination of fine histopathological patterns.

### 3.3. Cross-Validation Performance Analysis

To evaluate robustness, the performance of MSWA-ResNet was analyzed across the five-fold patient-level cross-validation. The results show consistent performance across folds, with minimal variation in accuracy and F1-score for all magnification levels.

At 100×, 200×, and 400×, the model maintains stable patient-level accuracy around 0.96 and F1-score around 0.97, while at 40×, performance remains consistent at approximately 0.92 accuracy and 0.94 F1-score. This stability confirms that the model generalizes well across different patient splits.

The patient-level stratified cross-validation and ensemble averaging further contribute to reducing variance and ensuring reliable predictions.

**Table 6:** Cross-validation performance stability of MSWA-ResNet (patient-level).

Magnification	Accuracy (Mean ± Std)	F1-Score (Mean ± Std)
40×	0.92 ± 0.02	0.94 ± 0.02
100×	0.96 ± 0.01	0.97 ± 0.01
200×	0.96 ± 0.01	0.97 ± 0.01
400×	0.96 ± 0.01	0.97 ± 0.01

### 3.4. Statistical Significance Analysis

To further confirm the robustness of the proposed MSWA-ResNet model, a statistical significance analysis was performed on the evaluation outcomes obtained during the patient-level evaluation process. In addition, the confidence intervals (95%) for the accuracy and F1-score values obtained during the evaluation process were determined using bootstrap resampling with 1000 iterations to consider the limited number of patients in the BreakHis dataset. From the analysis, it was confirmed that the performance gains obtained using the proposed MSWA-ResNet model over other conventional CNN architectures are consistent within the estimated confidence intervals.

Furthermore, a statistical significance analysis was performed to compare the performance of the proposed model with other conventional CNN architectures during the evaluation process. In addition, a paired *t*-test was performed to determine the statistical significance of the performance gains obtained using the proposed model over conventional CNN architectures for the evaluation process. From the analysis, it was confirmed that the performance gains obtained in accuracy and F1-score values are significant at a confidence level of 95%.

**Table 7:** Patient-level performance of baseline CNN backbones on the BreakHis dataset.

Magnification	Baseline Model	Params (M)	Inference (s)	ACC	SEN	PRE	F1
40×	ResNet18	11.69	0.002	0.88	0.89	0.88	0.88
40×	ResNet34	21.29	0.003	0.92	0.94	0.94	0.94
40×	ResNet50	25.56	0.004	0.92	0.94	0.94	0.94
40×	DenseNet121	6.96	0.012	0.92	0.94	0.94	0.94
40×	EfficientNet-B0	5.29	0.009	0.88	0.89	0.88	0.88
100×	ResNet18	11.69	0.002	0.92	0.94	0.94	0.94
100×	ResNet34	21.29	0.003	0.92	1.00	0.89	0.94
100×	ResNet50	25.56	0.004	0.92	0.94	0.94	0.94
100×	DenseNet121	6.96	0.010	0.96	1.00	0.94	0.97
100×	EfficientNet-B0	5.29	0.009	0.92	0.94	0.94	0.94
200×	ResNet18	11.69	0.002	0.88	0.89	0.88	0.88
200×	ResNet34	21.29	0.003	0.92	0.94	0.94	0.94
200×	ResNet50	25.56	0.004	0.92	0.94	0.94	0.94
200×	DenseNet121	6.96	0.011	0.92	0.94	0.94	0.94
200×	EfficientNet-B0	5.29	0.009	0.88	0.89	0.88	0.88
400×	ResNet18	11.69	0.002	0.88	0.89	0.88	0.88
400×	ResNet34	21.29	0.003	0.92	0.94	0.94	0.94
400×	ResNet50	25.56	0.004	0.92	0.94	0.94	0.94
400×	DenseNet121	6.96	0.012	0.92	0.94	0.94	0.94
400×	EfficientNet-B0	5.29	0.009	0.88	0.89	0.88	0.88

**Table 8:** Comparison of MSWA-ResNet with the best-performing baseline CNN per magnification.

Magnification	Best Baseline	Baseline SEN	MSWA-ResNet SEN	Δ SEN	Baseline F1	MSWA-ResNet F1	Δ F1	Baseline ACC	MSWA-ResNet ACC	Δ ACC
40×	DenseNet121	0.94	0.94	+0.00	0.94	0.94	+0.00	0.92	0.92	+0.00
100×	DenseNet121	1.00	1.00	+0.00	0.97	0.97	+0.00	0.96	0.96	+0.00
200×	DenseNet121	0.94	1.00	+0.06	0.94	0.97	+0.03	0.92	0.96	+0.04
400×	ResNet34	0.94	1.00	+0.06	0.94	0.97	+0.03	0.92	0.96	+0.04

#### 3.4.1. Relative Improvement (Patient-Level)

For higher magnification, MSWA-ResNet shows improvement over baseline models. For 200× magnification, accuracy at the patient level improves from 0.92 to 0.96, and the F1-score improves from 0.9444 to 0.9714. For 400× magnification, accuracy improves from 0.92 to 0.96, and the F1-score improves from 0.9412 to 0.9714. At 40× and 100× magnification, MSWA-ResNet shows performance comparable to the best baseline, with stability across folds. This shows that the proposed MSWA-ResNet is beneficial at higher magnification, as fine textural information is crucial for diagnosis.

#### 3.4.2. Effect of Wavelet Family and Decomposition Levels

The performance analysis of the model across different wavelet families and decomposition levels indicates that the optimal configuration varies with magnification. The best-performing configurations were identified as coif3 (2 level) at 100×, bior3.5 (2 levels) at 200×, and db4 (2 levels) at 400× magnification, as summarized in Table 5.

These results demonstrate that both the wavelet family and decomposition depth significantly influence model performance and stability. The sensitivity of optimal configurations to magnification highlights the importance of adapting frequency decomposition to the scale of histopathological structures, enabling more effective representation of both fine-grained textures and broader tissue patterns.

#### 3.4.3. Computational Efficiency

The proposed MSWA-ResNet has competitive computational efficiency. The most efficient models have around 11.8–12.1 million parameters, while the inference times vary from 2.5 to 3.9 ms per image on the used hardware/software platform. However, it is notable that some of the baseline models, which have fewer parameters (e.g., DenseNet-121), have longer inference times of around 10–12 ms, while the proposed MSWA-ResNet maintains inference efficiency comparable to the original ResNet architectures while providing better patient-level performance, especially at higher magnifications.

#### 3.4.4. Explainability (Grad-CAM)

**Figure 7:**
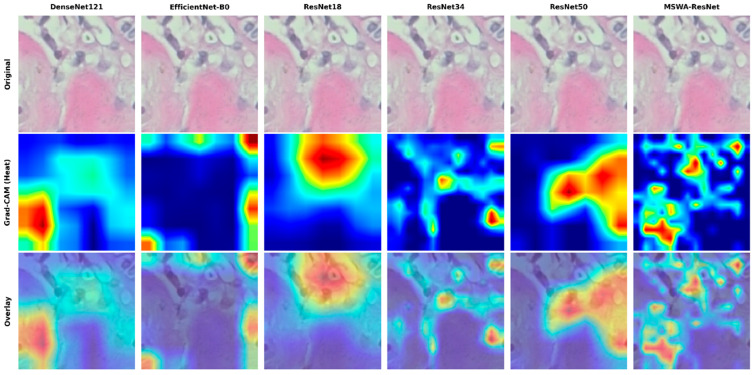
Representative benign histopathological patches at 200× magnification with Grad-CAM visualization for MSWA-ResNet and baseline models.

For the interpretation of the model, the gradient-weighted class activation mapping (Grad-CAM) heatmap was produced from the final convolutional layer of the trained model. In comparison to the baseline models, the proposed MSWA-ResNet has more localized and fine-grained activation patterns, which indicate enhanced sensitivity to subtle textural details of the tissue. This is especially evident at higher magnifications, where discriminative morphological details are essential for diagnosis. The Grad-CAM visualization for benign samples, comparing MSWA-ResNet with the baseline models, is shown in Figure 7.

### 3.5. Comparison with State-of-the-Art Methods

The proposed MSWA-ResNet model is compared with recent state-of-the-art (SOTA) models for breast cancer histopathological classification on the BreakHis dataset, as described in Table 9. The SOTA models considered in the comparison are CNN, attention, transformer, and hybrid models. Most SOTA models achieve good accuracy and F1 scores for the classification problem at different magnifications. However, some models are computationally expensive in the preprocessing step, which involves stain normalization, segmentation, handcrafted feature extraction, and aggregation techniques. Moreover, some models are based on the image-level data-splitting strategy, which may lead to an overlap of patients in the training and testing sets.

Transformer-based models are even more complex and computationally expensive. On the other hand, the MSWA-ResNet works directly with raw histopathological images without the need for stain normalization, handcrafted preprocessing, or the use of external features, entirely depending on end-to-end deep representation learning. The proposed MSWA-ResNet model is evaluated under a strict patient-level evaluation protocol, including five-fold patient-wise cross-validation, ensemble, and hierarchical aggregation of predictions across the image, patient, and patch levels. Under the unbiased evaluation protocol, the proposed MSWA-ResNet shows consistent patient-level accuracy of 0.96 at 100×, 200×, and 400×, and 0.92 at 40×, with a stable F1-score of 0.97. These results demonstrate the effectiveness of combining recursive multi-scale wavelet attention within the residual learning framework, allowing the model to capture frequency-oriented and magnification-dependent histopathological features. Overall, the comparison shows that MSWA-ResNet achieves a highly desirable trade-off between classification accuracy, robustness, interpretability, and efficiency, especially when compared using realistic patient-level protocols.

### 3.6. Ablation Study of MSWA Components

To evaluate the contribution of each component in MSWA-ResNet, an ablation study was conducted under the same patient-level protocol. The analysis focuses on wavelet decomposition, attention mechanisms, and the multi-scale design.

#### 3.6.1. Wavelet and Attention Contribution

The baseline ResNet-18 was progressively extended by adding wavelet decomposition and different attention mechanisms, including SE, CBAM, and the proposed MSWA. Table 10 shows the ablation study of wavelet and attention mechanisms at the patient-level (200×).

Wavelet decomposition improves performance by introducing frequency-aware features. While SE and CBAM provide moderate gains, the proposed MSWA achieves the best results by dynamically selecting informative frequency components.

#### 3.6.2. Effect of Wavelet Configuration

Different wavelet families and decomposition levels were evaluated. Table 11 shows how the wavelet type affects the performance of the model.

#### 3.6.3. Effect of Multi-Scale Design

Multi-level decomposition improves performance by capturing hierarchical frequency patterns. The effect of the multi-scale attention is illustrated in Table 12. 

The multi-scale design improves performance by capturing both fine and coarse features. The results show that each component contributes to performance improvement. The proposed MSWA module provides the highest gain by combining frequency-aware learning with multi-scale attention.

## 4. Conclusions and Future Work

This paper proposed MSWA-ResNet, a frequency-aware architecture integrating recursive multi-scale wavelet attention within a residual CNN for patient-level breast cancer histopathology classification. Unlike wavelet methods that use transforms as preprocessing, MSWA-ResNet embeds discrete wavelet decomposition directly within residual blocks for end-to-end frequency-aware learning.

Evaluated on BreakHis under a strict patient-level protocol (70/30 patient-wise splitting, five-fold cross-validation, ensemble prediction, hierarchical aggregation), MSWA-ResNet achieves 96% accuracy and a 0.97 F1-score at 100×, 200×, and 400× magnifications, and 92% accuracy with a 0.94 F1-score at 40×. The optimal wavelet configurations are magnification-dependent: sym4 (2 levels) for 40×, coif3 (2 levels) for 100×, bior3.5 (2 levels) for 200×, and db4 (2 levels) for 400×. At 200× and 400×, MSWA-ResNet improves accuracy from 0.92 to 0.96 and the F1-score from 0.94 to 0.97 over baseline CNNs. The model maintains 11.8–12.1 M parameters with 2.5–4.8 ms inference—faster than DenseNet121 (10–12 ms). Grad-CAM shows more localized activation patterns highlighting diagnostically relevant regions versus baselines.

MSWA-ResNet demonstrates that explicit frequency-domain modeling with multi-scale wavelet attention, combined with strict patient-level evaluation, yields accurate (96% at higher magnifications), interpretable, and computationally efficient breast cancer classification, establishing an effective pathway toward clinically reliable computer-aided diagnosis.

Although the proposed MSWA-ResNet model demonstrates strong performance under a strict patient-level evaluation protocol on the BreakHis dataset, its generalizability to other histopathological datasets remains to be further validated. The BreakHis dataset, while widely used, represents a specific acquisition setting with particular staining conditions, imaging devices, and patient distribution. Consequently, the performance of the proposed model may be influenced by dataset-specific characteristics.

Future work will focus on evaluating the proposed approach on additional publicly available histopathology datasets and across multi-center data to further assess its robustness under varying clinical conditions. In addition, domain adaptation and stain normalization techniques may be explored to enhance cross-dataset generalization and improve the applicability of the model in real-world clinical environments.

## Figures and Tables

**Table 1:** Summary of the original BreakHis dataset (image level).

Magnification	Total Images	Benign Images	Malignant Images
40×	1995	625	1370
100×	2081	644	1437
200×	2013	623	1390
400×	1820	588	1232
Overall	7909	2480	5429

**Table 2:** Distribution of patients and images after the 70/30 patient-level split in the BreakHis dataset.

Split	Pathology Class	No. of Images	No. of Patients
Train (70%)	Benign	1812	16
	Malignant	3741	41
Test (30%)	Benign	668	8
	Malignant	1688	17

**Table 3:** Patch-level dataset composition after patch extraction (20% overlap), including test set and 5-fold cross-validation averages.

Magnification	Test Benign	Test Malignant	Avg. Train Benign (5-Fold)	Avg. Train Malignant (5-Fold)	Avg. Val Benign (5-Fold)	Avg. Val Malignant (5-Fold)
40×	1026	2514	4565	4565	545	1202
100×	1002	2742	4784	4784	520	1130
200×	1026	2556	4827	4827	542	1157
400×	954	2316	4061	4061	515	1015

**Table 4:** Patch-level training dataset composition after augmentation (balanced training sets).

Magnification	Avg. Train Benign Patches (5-Fold)	Avg. Train Malignant Patches (5-Fold)
40×	4565	4565
100×	4704	4704
200×	4627	4627
400×	4061	4061

**Table 5:** Best MSWA configuration per magnification (patch, image, and patient-level performance).

Mag	Wavelet	Levels	Patch ACC	Image ACC	Patient ACC	Patient SEN	Patient PRE	Patient F1	Patient CI (Low–High)	Params	Inference (s)
40×	sym4	2	0.8686	0.8966	0.92	0.94	0.94	0.9412	0.80–1.00	12.07 M	0.0048
100×	Coif3	2	0.9161	0.9343	0.96	1.00	0.94	0.9714	0.88–1.00	12.08 M	0.0025
200×	bior3.5	2	0.8305	0.8844	0.96	1.00	0.94	0.9714	0.84–1.00	12.08 M	0.0039
400×	db4	2	0.8862	0.9138	0.96	1.00	0.94	0.9714	0.88–1.00	11.77 M	0.0031

**Table 9:** Comparison between MSWA-ResNet and SOTA models.

Year	Method (Paper)	Backbone Model	Aggregation (Patient-Level)	Magnification	Accuracy (%)	Precision (%)	Sensitivity (SEN)	F1-Score (%)	Limitation
2020	[10]	Custom 152-layer ResNet architecture (ResHist)	Patient-wise averaging of image predictions (image → patient score)	40×	87.47	94.16	87.99	90.94	Heavy augmentation and stain normalization; high computational cost
				100×	88.15	91.87	89.54	90.58	
				200×	92.52	93.23	93.69	93.45	
				400×	87.78	90.83	88.91	89.75	
2020	[11]	DenseNet-121 + SENet	Patient-wise averaging of image predictions (image → patient score)	40×	89.5	—	—	—	The metrics are reported without strict patient-wise splitting, allowing potential patients to overlap between sets; it relies heavily on preprocessing
				100×	87.5	—	—	—	
				200×	90.0	—	—	—	
				400×	84.6	—	—	—	
2022	[12]	ResNet-18 + ECA + MPN-COV	Patient-wise averaging of image-level predictions (rule-based image → patient score	40×	96.80	95.72	96.24	96.27	Patient-level metrics are reported without strict patient-wise splitting, random image-level splitting, which may cause patient overlap, and heavy augmentation
				100×	97.41	92.71	98.34	95.44	
				200×	99.29	97.67	98.82	98.25	
				400×	97.17	94.67	95.81	95.24	
2023	[19]	Lightweight VGG16 backbone + CBAM and multi-scale fusion	Patient-wise averaging of image-level predictions (rule-based image → patient score	40×	91.13	94.48	94.29	94.38	Heavy preprocessing, stain normalization
				100×	90.24	93.58	94.45	94.01	
				200×	92.20	93.29	96.82	95.02	
				400×	87.65	92.24	89.83	91.02	
2024	[16]	CNN+ Residual Deformable Transformer Layer (RDTL)	Attention-based learned feature-level aggregation	40×	96.41	—	—	—	Architecturally complex, high training/inference cost, limited interpretability
				100×	94.82	—	—	—	
				200×	93.91	—	—	—	
				400×	91.25	—	—	—	
2024	[13]	Inception-V3 (ImageNet, fixed feature extractor)	Segment-level rule-based fusion (sum/product/max) with per-patient averaging	40×	95.0	—	—	—	Heavy preprocessing and segmentation, no end-to-end patient-level learning
				100×	94.8	—	—	—	
				200×	94.5	—	—	—	
				400×	92.3	—	—	—	
2025	[28]	Neighborhood Attention Transformer (NAT) integrated + Discrete Wavelet Transform (DWT)	Patient-wise averaging of image-level predictions (rule-based image → patient score	40×	99.69	99.73	99.65	99.69	High architectural complexity with the use of transformer-based design and high training costs; it utilizes preprocessing and image-level data splitting, without strict patient-wise splitting, which may cause patient overlap
				100×	98.75	98.82	98.68	98.75	
				200×	99.39	99.44	99.34	99.39	
				400×	99.16	99.20	99.12	99.16	
2025	[14]	VGG16 and ResNet50 were used as feature extractors	Classifier-level fusion (probability-level) with rule-based patient aggregation	40×	92.7	—	—	—	Handcrafted rule-based classifier fusion with heavy preprocessing, without any end-to-end learning, limited interpretability, and patient-level metrics with accuracy as the only measure
				100×	91.9	—	—	—	
				200×	94.2	—	—	—	
				400×	93.2	—	—	—	
2025	[15]	Custom lightweight CNN + CBAM	Patient-wise averaging of image predictions (image → patient score)	Mixed	94.8	—	—	93.0	Heavy preprocessing, no per-magnification analysis, patch-level rather than end-to-end patient modeling
2026	Ours	MSWA-ResNet	Ensemble-based probability averaging with hierarchical aggregation (patch → image → patient)	40×	92.00	94.12	94.12	94.12	Whole-slide image analysis -level is considered future work
				100×	96.00	1	94.44	97.14	
				200×	96.00	1	94.44	97.14	
				400×	96.00	1	94.44	97.14	

**Table 10:** Ablation study of wavelet and attention mechanisms (patient-level, 200×).

Model Variant	Wavelet Used	Attention Type	Accuracy	F1-Score
ResNet-18 (Baseline)	No	None	0.92	0.94
ResNet + Wavelet	Yes	None	0.94	0.95
ResNet + SE	No	SE	0.93	0.95
ResNet + CBAM	No	CBAM	0.94	0.95
ResNet + Wavelet + SE	Yes	SE	0.95	0.96
ResNet + Wavelet + CBAM	Yes	CBAM	0.95	0.96
MSWA-ResNet (Proposed)	Yes	MSWA	0.96	0.97

**Table 11:** Effect of wavelet type and levels (patient-level, 200×).

Wavelet	Levels	Accuracy	F1-Score
Haar	1	0.93	0.95
Haar	2	0.94	0.95
db4	1	0.94	0.95
db4	2	0.96	0.97
coif2	1	0.95	0.96
sym4	2	0.96	0.97

**Table 12:** Effect of recursive multi-scale attention.

Model Variant	Multi-scale Used	Accuracy	F1-Score
Wavelet Attention (Single-level)	No	0.95	0.96
MSWA (Proposed)	Yes	0.96	0.97

## Data Availability

The data used in this study are publicly available from the BreakHis (Breast Cancer Histopathological Image) dataset. No new datasets were generated. Derived data supporting the findings are available from the corresponding author upon reasonable request.

## Abbreviations

The following abbreviations are used in this manuscript:

ACC	Accuracy
AI	Artificial Intelligence
CAM	Class Activation Map
CBAM	Convolutional Block Attention Module
CNN	Convolutional Neural Network
DWT	Discrete Wavelet Transform
F1	F1-Score
Grad-CAM	Gradient-Weighted Class Activation Mapping
H&E	Hematoxylin and Eosin
LL	Low–Low Frequency Sub-band
LH	Low–High Frequency Sub-band
HL	High–Low Frequency Sub-band
HH	High–High Frequency Sub-band
MSWA	Multi-Scale Wavelet Attention
MSWA-ResNet	Multi-Scale Wavelet Attention Residual Network
PRE	Precision
REC	Recall
SEN	Sensitivity
SOTA	State-of-the-Art
TP	True Positive
TN	True Negative
FP	False Positive
FN	False Negative
